# Comparison of Two Sampling Techniques for Evaluating Ruminal Fermentation and Microbiota in the Planktonic Phase of Rumen Digesta in Dairy Cows

**DOI:** 10.3389/fmicb.2020.618032

**Published:** 2020-12-23

**Authors:** Camila Flavia de Assis Lage, Susanna Elizabeth Räisänen, Audino Melgar, Krum Nedelkov, Xianjiang Chen, Joonpyo Oh, Molly Elizabeth Fetter, Nagaraju Indugu, Joseph Samuel Bender, Bonnie Vecchiarelli, Meagan Leslie Hennessy, Dipti Pitta, Alexander Nikolov Hristov

**Affiliations:** ^1^The Pennsylvania State University, University Park, PA, United States; ^2^Department of Clinical Studies-New Bolton Center, School of Veterinary Medicine, University of Pennsylvania, Kennett Square, PA, United States

**Keywords:** non-invasive sampling techniques, stomach tube method, rumen microbiome, rumen cannula, rumen fermentation, stomach tube

## Abstract

The objective of this experiment was to compare ruminal fluid samples collected through rumen cannula (RC) or using an oral stomach tube (ST) for measurement of ruminal fermentation and microbiota variables. Six ruminally cannulated lactating Holstein cows fed a standard diet were used in the study. Rumen samples were collected at 0, 2, 4, 6, 8, and 12 h after the morning feeding on two consecutive days using both RC and ST techniques. Samples were filtered through two layers of cheesecloth and the filtered ruminal fluid was used for further analysis. Compared with RC, ST samples had 7% greater pH; however, the pattern in pH change after feeding was similar between sampling methods. Total volatile fatty acids (VFA), acetate and propionate concentrations in ruminal fluid were on average 23% lower for ST compared with RC. There were no differences between RC and ST in VFA molar proportions (except for isobutyrate), ammonia and dissolved hydrogen (dH_2_) concentrations, or total protozoa counts, and there were no interactions between sampling technique and time of sampling. Bacterial ASV richness was higher in ST compared with RC samples; however, no differences were observed for Shannon diversity. Based on Permanova analysis, bacterial community composition was influenced by sampling method and there was an interaction between sampling method and time of sampling. A core microbiota comprised of *Prevotella*, *S24-7*, unclassified *Bacteroidales* and unclassified *Clostridiales*, *Butyrivibrio*, unclassified *Lachnospiraceae*, unclassified *Ruminococcaceae*, *Ruminococcus*, and *Sharpea* was present in both ST and RC samples, although their relative abundance varied and was influenced by an interaction between sampling time and sampling method. Overall, our results suggest that ruminal fluid samples collected using ST (at 180 to 200 cm depth) are not representative of rumen pH, absolute values of VFA concentrations, or bacterial communities >2 h post-feeding when compared to samples of ruminal fluid collected using RC. However, ST can be a feasible sampling technique if the purpose is to study molar proportions of VFA, protozoa counts, dH_2_, and ammonia concentrations.

## Introduction

Sampling and analysis of ruminal fluid are important tools in ruminant nutrition research and in addition to studies focused on ruminal fermentation, there is an increasing interest in understanding ruminal microbiota. Objectives have been to test strategies in ruminal microbiota manipulation toward a more efficient fermentation and possibly mitigate enteric methane emissions. A better understanding of individual variations in ruminal microbial population may also help identify, and possibly select for, more efficient animals.

Rumen cannulation (RC) or stomach tubing (ST) are the two main techniques used to study ruminal fermentation and microbial community composition (Ramos-Morales et al., 2014). Collecting ruminal contents through RC is the standard method for rumen sampling (Nocek, 1997). However, since cannulation is an expensive and invasive method, it is usually performed on a small number of animals. The use of ST allows ruminal fluid collection in intact animals, increasing the possibility of studies with more animals or studies for genetic selection purposes.

The main challenges of the ST technique are contamination of the rumen sample with saliva and unrepresentative sampling of whole ruminal contents (Shen et al., 2012). Although there are reports comparing both RC and ST techniques for fermentation variables, discrepancies exist between studies on how comparable the two techniques are in relation to pH and VFA. Some studies have reported significant differences in these parameters (Geishauser and Gitzel, 1996; Duffield et al., 2004; Wang et al., 2016), whereas others did not see differences between the two techniques (Geishauser and Gitzel, 1996; Shen et al., 2012).

Several factors can affect fermentation variables and microbial community of dairy cows, including breed, age, and physiological conditions. In situations where similar animals are compared, dietary factors such as type of diet fed, feed intake, and time of sampling will have a significant influence on ruminal fermentation variables and microbiota (Stewart et al., 1958). The intensity of ruminal fermentation varies throughout the day (Li et al., 2009) and little information is available on how fermentation and microbiota data obtained using the RC and ST sampling techniques are affected by time of sampling relative to feeding when similar animals in similar dietary conditions are compared. Findings of Li et al. (2009) demonstrated that the distribution of detectable bacteria was relatively stable among different locations within the rumen, but the quantity of individual bacterial species changed throughout the day in response to feeding. Ramos-Morales et al. (2014) compared samplings using ST as an alternative to RC and observed similarities in rumen microbiota between techniques. These authors, however, reported differences in microorganism distribution pre- and post-feeding.

Stomach tubing recovers samples mostly from the planktonic phase with small finely digested particles and does not permit sampling from different sites within the rumen, and therefore samples acquired using this method may have a different distribution of microbial communities compared to rumen contents collected via cannula (Firkins and Yu, 2015). While Henderson et al. (2013) compared microbial diversity in whole rumen contents collected using both RC and ST methods, Paz et al. (2016) added portions of solid particles to the planktonic phase collected using ST to represent both ruminal fractions (Paz et al., 2016). The liquid fraction is typically used to measure fermentation variables but there are no reports comparing microbial populations in the liquid fraction of rumen contents collected by different methods. Therefore, the objective of this study was to compare rumen fermentation parameters and ruminal microbiota composition between RC and ST techniques and to investigate if time post-feeding can affect differences between the two techniques. We hypothesized that distribution of ruminal microbiota and fermentation patterns would differ between sampling techniques and these differences may be affected by sampling time post-feeding.

## Materials and Methods

### Animals and Diets

Six cannulated lactating Holstein cows [averaging (± SD) 49.8 ± 9.68 kg/d milk yield; 2.20 ± 0.37 lactations; 565 ± 40.9 kg BW; and 45.9 ± 11.7 days in milk at the beginning of the experiment] were used in the study. The cows were housed in the tie-stall barn of The Pennsylvania State University’s Dairy Teaching and Research Center for 17 days before the experiment began. They were fed once daily (at 0800 h) a standard diet containing (DM basis): 36.8% corn silage, 15.2% alfalfa haylage, 13.6% ground corn grain, 8.8% canola meal, 8.0% bakery by-product meal, 5.6% roasted soybean seeds, 4.8% molasses, 3.2% whole cottonseed, 2.0% grass hay, 1.8% vitamin and mineral premix, and 0.2% of a slow-release urea source (Optigen^®^, Alltech Inc., Nicholasville, KY, United States). The analyzed composition of the basal diet was as follows (DM basis): 15.0% CP, 29.8% NDF, 19.1% ADF, 4.7% ether extract, 6.12% ash, and estimated 6.15 MJ/kg of net energy for lactation and 47.1% non-fiber carbohydrates. Chemical analyses of the total mixed ration (TMR) were conducted at Cumberland Valley Analytical Services (Waynesboro, PA, United States) and non-fiber carbohydrate and net energy for lactation were estimated based on NRC (2001).

The diet was fed as a TMR to achieve about 10% refusals. Cows were milked twice daily at 0600 and 1800 h and had free access to drinking water. Before the morning milking, cows were kept in an exercise area for 1 h.

### Experimental Design and Sampling Methods

The rumen sampling occurred over two consecutive days at 0, 4, and 8 h after feeding on day one and 2, 6 and 12 h after feeding on day two. For RC sampling, the cannula lid was removed and whole ruminal content samples were collected with a gloved hand from four locations in the rumen: the ventral sac, the atrium or reticulum, and two samples from the feed mat. Approximately 200 g of contents were collected from each location; contents were thoroughly mixed, and a subsample was used for further processing and analyses.

The ST sampling device consisted of a 244-cm long polyvinyl chloride orogastric tubing with a 15-mL perforated plastic conical tube attached to one end acting as a sieve in the rumen. The other end of the ST was attached to an electric vacuum pump (Gast model 0823-v13q-g608nex, Septic solutions Inc^®^, Dieterich, IL, United States) with 68.9 kPa of maximum continuous pressure. During a sampling event, the head of the animal was restrained, and ruminal fluid was collected by passing the tubing using an oral speculum down the esophagus into the rumen. The tubing was gently pushed through the rumen mat to collect ruminal contents. Approximately 180 to 200 cm of the ST was in the cow with the remainder being outside of the cow, thus providing the flexibility to move the tube and extract ruminal fluid. Approximately 200 mL of initially sampled ruminal fluid was discarded due to possible saliva contamination. After discarding the initial volume, an additional 500 mL of ruminal fluid were collected and processed for further analyses.

### Processing and Chemical Analysis of Samples

Whole ruminal contents from RC and predominantly ruminal fluid from ST were filtered through two layers of cheesecloth to separate fluid and solids. Studies have used two layers (Martinez-Fernandez et al., 2019), four layers (Pitta et al., 2010; Ji et al., 2017), and eight layers (Craig et al., 1987) of cheesecloth to separate ruminal fractions; however, Firkins et al. (2020) pointed out that maximum two layers of cheesecloth should be used if evaluating protozoa since larger protozoa can be entrapped and under-represented using multiple (i.e., more than 2) layers.

Subsamples of the ruminal fluid were immediately analyzed for pH (pH meter 59000–60 pH Tester, Cole-Parmer, Instrument Company, Vernon Hills, IL, United States) and processed for analyses of ammonia and volatile fatty acids (VFA) as described in Hristov et al. (2011). Samples for protozoal enumeration were preserved (Hristov et al., 2011) and counted according to standard procedures (Dehority, 1993). A separate sample was processed for dissolved hydrogen (dH_2_), which is a variable of interest in methane enteric mitigation studies, according to Wang et al. (2014).

### Microbial Analysis

Ruminal fluid samples were aliquoted for DNA analysis and immediately frozen at -80°C until further analysis. The ruminal fluid samples were extracted for genomic DNA using the “Repeated Bead Beating and Column” (RBB + C) purification method followed by extraction with the QIAmp Fast DNA Stool Mini Kit (Qiagen Sciences, Germantown, MD, United States; Yu and Morrison, 2004). The extracted DNA was PCR-amplified using the bacterial-specific primers BSF8 (27F) and BSR357 annealing to the V1–V2 region of the 16S rRNA bacterial gene as described in Pitta et al. (2014). Polymerase chain reaction was performed in triplicate using the Accuprime Taq DNA Polymerase System (Invitrogen, Carlsbad, CA, United States). The thermal cycling conditions and purification of PCR libraries were performed as described in Pitta et al. (2014). The amplicons generated for each sample were pooled in equimolar concentration and sequenced using the MiSeq Illumina Platform (Illumina Inc., San Diego, CA, United States).

### Bioinformatics and Data Analysis

Microbiota bioinformatics were performed with QIIME2-2018.4 (Bolyen et al., 2019). Raw sequence data were demultiplexed using the q2-demux plugin followed by quality filtering, denoising and assigned to amplicon sequence variants (ASV) with DADA2 (Callahan et al., 2016) (via q2-dada2) following the parameters: the input sequences reads were truncated at the 3 frame end of the sequence at 230 nucleotides and default settings were used for the remaining options of this plugin. The ASV were aligned with mafft (Katoh et al., 2002) (via q2-alignment) and used to construct a phylogeny with fasttree2 (Price et al., 2010) (via q2-phylogeny) with default settings.

Alpha-diversity estimates (observed ASV and Shannon diversity) were calculated as per methods described in Pitta et al. (2014). In addition, phylogenetic based alpha diversity (Faith’s phylogenetic diversity) was performed. Beta diversity metrics (weighted UniFrac), and the Principle Coordinate Analysis (PCoA) were performed in R according to methods described in Pitta et al. (2014). Taxonomy was assigned to ASV using the q2-feature-classifier (Bokulich et al., 2018) classify-sklearn naïve Bayes taxonomy classifier against the Greengenes (version 13_8) (McDonald et al., 2012). The measured alpha diversity matrices were compared between the sampling techniques using Wilcoxon rank sum test. A non-parametric permutational multivariate ANOVA (PERMANOVA) test, implemented in the vegan (Anderson, 2001) package for R, was used to test the effects (sampling method, time, and the interaction of sampling method × sampling time with 999 permutations) on overall community composition weighted UniFrac distance. To reproduce PERMANOVA results we used seed setting of “1234.” The raw read counts from the 16S rRNA ASV abundance table were collapsed at taxonomic rank and compositionally normalized (relative abundance) such that each sample sums to 100.

### Statistical Analysis

Rumen fermentation data were analyzed using the MIXED procedure of SAS (release 9.4, SAS Institute Inc., Cary, NC, United States). The model contained sampling method, sampling time, and sampling method × sampling time interaction. Cow within sampling technique was considered random effect. Data were analyzed as repeated measures using the ar(1) covariance structure. Rumen microbiota data were analyzed using the GLIMIX procedure of SAS. The model contained sampling method, sampling time, and sampling method × sampling time interaction, with the beta distribution option. The RANDOM statement contained intercept with cow within sampling method as subject. All data are presented as least squares means. Significance was declared at *P* ≤ 0.05 and tendency was declared at 0.05 < *P* ≤ 0.10.

## Results

**FIGURE 1:**
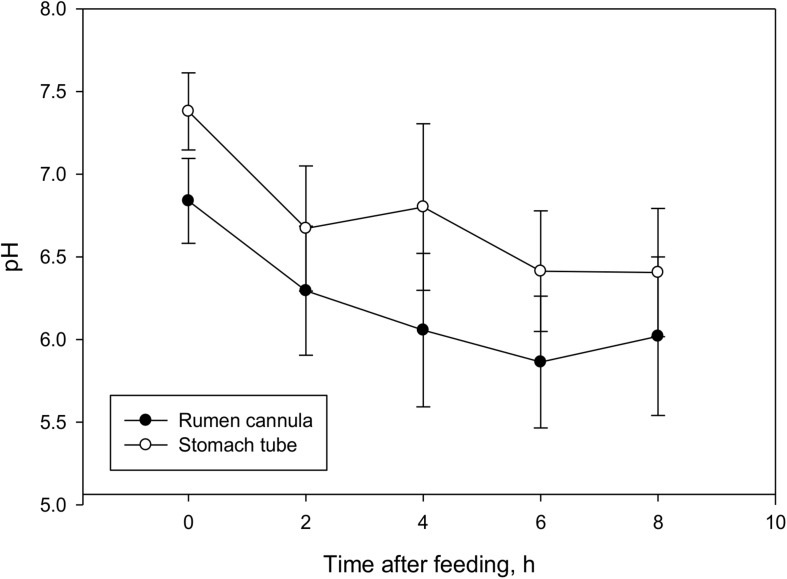
Effect of sampling techniques (rumen cannula vs stomach tube) on pH in lactating dairy cows (means ± SE; *n* = 66). Overall sampling method effect, *P* < 0.001; time of sampling effect, *P* < 0.001; sampling method × time of sampling interaction, *P* = 0.66.

**FIGURE 2:**
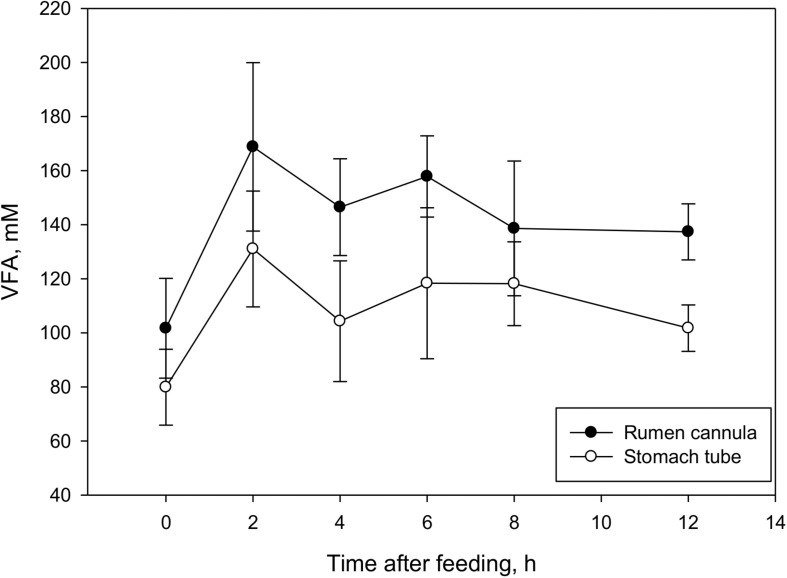
Effect of sampling techniques (rumen cannula vs stomach tube) on total volatile fatty acid (VFA) concentration (m*M*) in lactating dairy cows (means ± SE; *n* = 69). Overall sampling method effect, *P* < 0.001; time of sampling effect, *P* < 0.001; sampling method × time of sampling interaction, *P* = 0.44.

Ruminal fluid collected through ST had a pH on average 0.47 points greater (*P* < 0.001, Table 1) than ruminal fluid collected through RC, and the difference between the two sampling techniques persisted throughout the course of sampling (Figure 1). In addition to a greater pH, ST ruminal fluid also had lower (*P* = 0.002) total VFA concentration, lower acetate and propionate concentrations (*P* < 0.001 and *P* = 0.04, respectively), and tended to have lower butyrate and isovalerate concentrations (*P* = 0.06 and *P* = 0.08, respectively). No differences in concentrations of isobutyrate and valerate were observed between the two techniques. Total VFA concentrations had similar patterns for both sampling methods over the course of sampling (Figure 2). No differences in molar proportions of VFA were observed between the two sampling techniques, except for a slightly greater (*P* = 0.03) proportion of isobutyrate in ruminal fluid collected via ST in comparison to RC. There were no differences (*P* ≥ 0.11) in ammonia or dH_2_ concentrations and protozoal counts between sampling methods.

**TABLE 1:** Effect of sampling techniques (rumen cannula, RC, vs stomach tube, ST) on rumen fermentation variables in lactating dairy cows.

Item	Sampling method		SEM^1^	*P*-value^2^
	RC	ST		
pH	6.27	6.74	0.096	<0.001
Total VFA, m*M*	142	109	5.58	0.002
Acetate	82.2	64.2	2.50	< 0.001
Propionate	37.5	28.4	2.66	0.04
Isobutyrate	0.40	0.37	0.054	0.67
Butyrate	16.4	12.3	1.35	0.06
Isovalerate	1.60	1.26	0.124	0.08
Valerate	3.65	2.39	0.567	0.15
VFA,% of total VFA				
Acetate	58.5	59.4	1.35	0.64
Propionate	26.3	25.8	1.29	0.77
Isobutyrate	0.30	0.37	0.055	0.03
Butyrate	11.3	11.2	0.83	0.91
Isovalerate	1.13	1.19	0.098	0.70
Valerate	2.47	2.10	0.373	0.50
Acetate: propionate	2.26	2.35	0.150	0.70
Ammonia, m*M*	4.96	5.22	0.680	0.78
Total protozoa^3^, × 10^4^/mL	25.1	8.55	6.08	0.11
Dissolved hydrogen, μ*M*	3.01	2.34	0.881	0.33

*^1^Highest SEM shown; VFA, *n* = 69; pH, *n* = 66; dissolved hydrogen, *n* = 57; protozoa, *n* = 58 (*n* represents number of observations used in the statistical analysis).^2^Main effect of sampling method; time of sampling, *P* < 0.001; sampling method × time of sampling, *P* ≥ 0.28.^3^Actual protozoal counts were log10-transformed for the statistical analysis.*

The effect of time of sampling was significant (*P* ≤ 0.04) for all fermentation variables studied (Figures 1, 2), except for a tendency in dH_2_ concentration (*P* = 0.07) and no effects on total protozoal counts (*P* = 0.25). There were no interactions between sampling technique and sampling time (*P* ≥ 0.31) for any of the analyzed fermentation variables.

**FIGURE 3:**
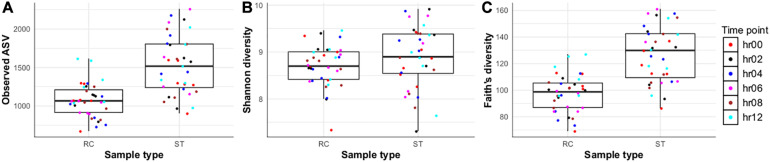
**(A–C)** Comparison of alpha diversity of liquid fraction from samples collected using ruminal cannula (RC) and stomach tube (ST) at different sampling times relative to feeding [before feeding (hr00); 2 h after feeding (hr02), 4 h after feeding (hr04); 6 h after feeding (hr06); 8 h after feeding (hr08); and 12 h after feeding (hr12)] for **(A)** Species richness and **(B)** Shannon diversity **(C)** Faith’s phylogenetic diversity.

**FIGURE 4:**
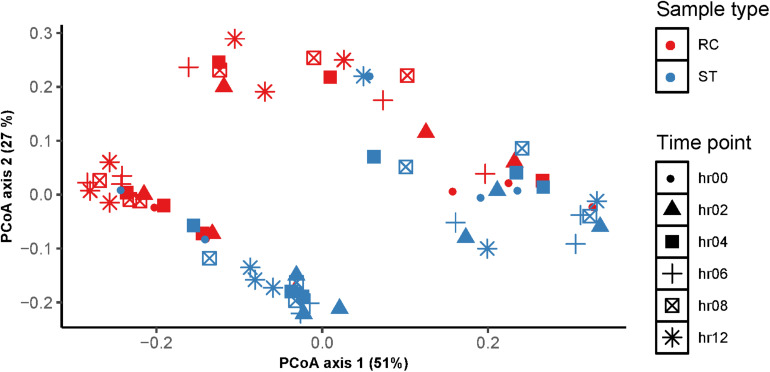
Comparison of bacterial community composition of liquid fraction from samples collected using stomach tube (ST) and ruminal cannula (RC) at different sampling time [before feeding (hr00); 2 h after feeding (hr02), 4 h after feeding (hr04); 6 h after feeding (hr06); 8 h after feeding (hr08) and 12 h after feeding (hr12)]. The principal coordinate (PC) plots show weighted pairwise UniFrac distances between samples.

At the bacterial community level, species richness in ruminal fluid samples was different (Wilcoxon test; *P* ≤ 0.05) between sampling methods with greater species richness in ruminal fluid collected through ST compared with RC (Figure 3A). Further, differences between ST and RC ruminal fluid were observed at 4 and 6 h after feeding, but not at other sampling times. WhileShannon diversity index values did not show differences, Faith’s phylogenetic diversity showed differences between ST and RC samples (Figures 3B,C). At the community level, there were differences between RC and ST bacterial communities (Figure 4). The Permanova analysis revealed that clustering of bacterial communities was influenced by sampling method (*P* ≤ 0.05) but not time (*P* ≤ 0.62), and the interaction of sampling method × sampling time (*P* ≤ 0.44). Pairwise comparisons indicated differences between ST and RC samples at 6, 8, and 12 h, but not at 0, 2, or 4 h after feeding.

At the phylum level, the most dominant bacterial phyla identified in the study was *Firmicutes* followed by *Bacteroidetes*. The mean values for the relative abundance of *Firmicutes* in ST samples were lower (*P* = 0.01) when compared to those of RC samples (51.3 and 67.6% for ST and RC, respectively). The mean relative abundance of *Bacteroidetes* in ST samples was greater (*P* = 0.004) compared to RC samples (43.5 and 26.7% for ST and RC, respectively).

The most abundant individual microbial populations (>0.1% of relative abundance) are presented in Table 2. Among *Bacteroidetes*, the most abundant genera were *Prevotella* followed by *S24-7* and unclassified *Bacteroidales*. In *Firmicutes*, the most abundant genera were unclassified *Clostridiales*, *Butyrivibrio*, unclassified *Lachnospiraceae*, unclassified *Ruminococcaceae*, *Ruminococcus*, *Sharpea*, and several others that had greater than 1% relative abundance. The relative abundance of the individual microbial populations varied and was influenced by sampling method, sampling time, and their interactions (Table 2). A majority of bacterial populations including the most abundant rumen bacterial populations, such as *Prevotella* and *Prevotellaceae* from *Bacteroidetes* and several genera from *Firmicutes* including *Bulleidia*, *Butyrivibrio*, unclassified *Clostridiales*, *Clostridium*, unclassified *Lachnospiraceae*, *Mogibacteriaceae*, unclassified *Ruminococcaceae*, *Shuttleworthia*, *Succiniclasticum*, and *Veillonellaceae*, were all influenced (*P* ≤ 0.05) by an interaction between sampling method and sampling time.

**TABLE 2:** Effect of sampling techniques (rumen cannula, RC, vs stomach tube, ST) on bacterial composition (%)^1^ in lactating dairy cows.

Bacterial taxa	Sampling method		SEM^2^	*P*-value		
	RC	ST		TR	T	TR × T
*Actinobacteria* (phylum)						
*Coriobacteriaceae* (family)	2.41	1.31	0.239	0.002	0.03	0.01
*Corynebacterium* (genus)	0.18	0.11	0.031	0.10	0.07	0.16
*Unclassified bacteria* (phylum)	0.44	0.37	0.064	0.42	0.63	0.65
*Bacteroidetes* (phylum)						
*Bacteroidales* (order)	2.41	2.25	0.309	0.71	0.18	0.23
*CF231* (genus)	0.39	0.41	0.106	0.85	0.07	0.22
*Paraprevotellaceae* (family)	0.63	0.92	0.074	0.01	0.22	0.07
*Prevotella* (genus)	17.6	34.6	4.39	0.01	0.02	<0.001
*Prevotellaceae* (family)	0.44	0.78	0.095	0.01	0.06	0.04
*S247* (family)	2.75	2.08	0.200	0.03	0.14	0.60
*YRC22* (genus)	0.33	0.52	0.075	0.06	0.13	0.53
*Cyanobacteria* (phylum)						
YS2 (genus)	0.03	0.14	0.035	0.004	0.06	0.01
*Fibrobacteres* (phylum)						
*Fibrobacter* (genus)	0.04	0.13	0.032	0.01	0.09	0.01
*Firmicutes* (phylum)						
*Bulleidia* (genus)	1.93	1.06	0.736	0.30	<0.001	<0.001
*Butyrivibrio* (genus)	9.59	4.86	0.983	0.001	0.05	<0.001
*Christensenellaceae* (family)	0.29	0.16	0.101	0.28	0.02	<0.001
*Clostridiales* (order)	18.1	11.9	1.14	0.001	0.02	<0.001
*Clostridium* (genus)	0.13	0.17	0.062	0.64	0.003	0.001
*Coprococcus* (genus)	1.47	1.42	0.212	0.87	0.98	0.02
*Dialister* (genus)	0.21	0.32	0.247	0.71	<0.001	0.001
*Eubacterium* (genus)	0.34	0.19	0.102	0.18	0.06	0.002
*L7A-E11* (genus)	0.10	0.07	0.022	0.18	0.94	0.82
*Lachnospiraceae* (family)	8.92	5.76	0.300	<0.001	0.17	0.001
*Lactobacillus* (genus)	0.07	0.12	0.036	0.24	0.001	0.25
*Mogibacteriaceae* (family)	1.54	1.15	0.113	0.02	0.41	0.004
*Moryella* (genus)	0.43	0.28	0.038	0.01	0.02	0.20
*Oscillospira* (genus)	0.11	0.13	0.050	0.77	0.98	0.87
*P75a5* (genus)	0.14	0.09	0.027	0.13	0.97	0.30
*Pseudobutyrivibrio* (genus)	0.06	0.11	0.033	0.21	0.54	0.03
*RFN20* (genus)	0.22	0.26	0.029	0.30	0.25	0.06
*Ruminococcaceae* (family)	6.36	3.83	0.528	0.002	0.24	< 0.001
*Ruminococcus* (genus)	3.85	5.23	2.334	0.64	0.05	0.88
*Selenomonas* (genus)	0.09	0.20	0.067	0.15	<0.001	0.09
*Sharpea* (genus)	3.28	4.37	0.611	0.18	0.01	0.47
*Shuttleworthia* (genus)	1.03	1.21	0.274	0.63	0.12	0.01
*Streptococcus* (genus)	0.08	0.10	0.014	0.21	0.05	0.30
*Succiniclasticum* (genus)	1.55	1.39	0.385	0.75	<0.001	0.04
*Veillonellaceae* (family)	0.36	0.36	0.092	0.98	<0.001	0.001
*Weissella* (genus)	0.11	0.22	0.052	0.06	0.002	0.74
*Proteobacteria* (phylum)						
*Succinivibrionaceae* (family)	0.14	0.34	0.048	0.001	0.18	0.11
*Spirochetes* (phylum)						
*Treponema* (genus)	0.12	0.21	0.059	0.15	<0.001	0.03
*SR1* (phylum)	0.06	0.11	0.048	0.31	0.42	0.07
*Tenericutes* (phylum)						
*RF39* (family)	0.67	0.74	0.168	0.76	0.05	0.03
*TM7* (phylum)						
*F16* (family)	0.56	0.57	0.102	0.97	0.18	0.06

*^1^The percentage represents the percentage of the total sequences analyzed within the sample. ^2^Largest SEM published in table; *n* = 68 for all variables (*n* represents the number of observations used in the statistical analysis).*

## Discussion

The purpose of this study was to compare the fermentation variables and microbial community composition in the liquid fraction of ruminal digesta samples collected using ST and RC at multiple timepoints post-feeding in the rumen of dairy cows. The study showed differences in pH and total and individual VFA concentrations but not in acetate: propionate ratio, dH_2_ or ammonia concentrations, molar proportions of VFA, or protozoa counts between the two methods. The greater pH of ruminal fluid for ST versus RC samples observed in the current experiment is in agreement with Duffield et al. (2004), who reported differences of 0.44 and 0.34 pH units (without or with discarding the first 200 mL of the ruminal sample, respectively) for ruminal fluid obtained with ST compared with RC. Similar findings were also reported by Terré et al. (2013) even when the first aliquot of ruminal fluid with visual saliva contamination was discarded, indicating that salivary contamination may not be responsible for the differences between the two methods. Differences in pH and VFA between ST and RC have been associated with sampling depth in the rumen (Bryant, 1964; Shen et al., 2012); these authors reported differences in fermentation variables in RC and ST when the ST probe was inserted to a depth of 180 cm, but these differences disappeared when the insertion depth was increased to 200 cm. A recent study (Larsen et al., 2020) compared samples obtained from a modified version of ST called the ororuminal FLORA sampling device to RC samples, but the RC method used in this study involved a suction strainer inserted into the ventral sac via the cannula. An increase in pH and a decrease in VFA concentrations, similar to the findings of the current study, were reported and the authors concluded that resistance from firm digesta prevents the ST from passing beyond the cranial or dorsal rumen into the ventral rumen. Collectively, it may be inferred that differences in sampling location of the rumen but not the sampling techniques may explain differences in the ruminal parameters. In the current study, even though ruminal fluid concentrations of individual VFA were markedly lower for ST samples compared with RC, molar proportions of VFA in ST and RC ruminal fluid samples were similar for most VFA. Similar results have been reported by others (Li et al., 2009; Shen et al., 2012; Wang et al., 2016; Ma et al., 2018). In agreement with data from the current experiment, Terré et al. (2013) reported that VFA molar proportions for ST and RC ruminal fluid were all highly correlated, suggesting that ST can be a feasible technique if the purpose of the experiment is to evaluate differences in molar proportions of VFA. In addition, in the present experiment acetate: propionate ratio was similar between ST and RC samples which differ from results observed in Wang et al. (2016).

The effect of time of sampling was significant for almost all variables studied in the current experiment. Increased availability of fermentable substrate after feeding increases fermentation rate, subsequently leading to an increase in VFA production and concentration, which in turn causes a drop in pH (Allen, 1997). In the current study, a greater rumen pH value before feeding was observed which decreased steadily until 6 h post-feeding before starting to increase again. Although VFA concentration at 2 h was greater (*P* < 0.001; across treatments) than at 4 h, total VFA concentration peaked again at 6 h (which coincided with the lowest pH, and there was no difference (*P* = 0.08) between 2 and 6 h. These trends are typically observed in ruminants fed once daily (Soto-Navarro et al., 2000). There were no interactions between sampling technique and sampling time for any of the analyzed fermentation variables indicating that results observed for the two sampling techniques remained consistent over time, which is in agreement with previous work by Wang et al. (2016).

Data presented in the current experiment indicate that there were differences in bacterial communities, particularly the relative abundance of individual bacterial populations, between ST and RC which, unlike the fermentation data, were driven predominantly by an interaction between sampling method and sampling time. Studies (Ramos-Morales et al., 2014; Song et al., 2018) have compared microbial profiles using ST and RC samples and observed no differences between sampling techniques. These reports relied only on one sampling time from each animal and fiber or digesta particles were included in the ST samples. Furthermore, different fractions of rumen contents (fiber-adherent fraction and liquid fraction) had different microbial composition (Pitta et al., 2010; De Mulder et al., 2017). In the current study, we intentionally analyzed only the liquid fraction from both sampling methods which revealed that there were differences in bacterial communities in the liquid fraction of RC and ST samples. Deusch et al. (2017) reported significant variations in community composition and functional profiles of microbiota sampled from different locations (micro-environments) within the rumen which agrees with findings in the current study. Overall, the microbial analysis at the community level revealed no differences between RC and ST ruminal fluid samples collected at 0 and 2 h post-feeding, indicating that bacterial communities retrieved using the two methods were similar before feeding and soon after the cows started consuming the feed, compared to samples collected >2 h post-feeding. An experiment conducted at The Pennsylvania State University evaluating the effects of feeding time and feeding frequency on feeding behavior of dairy cows reported that cows fed once a day at 0830 h ate 22.6% of their TMR in the first two hours post-feeding (Niu et al., 2014). Based on data from the current study, it can be concluded that ruminal microbiota, at the community level, were similar between RC and ST (despite different sampling locations) before feeding; however, introducing feed into the rumen had a significant influence on the diversity and distribution of bacterial communities. This indicates that feed intake and feeding behavior may lead to redistribution of bacterial populations in different locations to allow fermentation, thus explaining the differences in microbial communities between RC and ST >2 h after feeding.

A greater relative abundance of *Bacteroidetes* and a lower relative abundance of *Firmicutes* in ruminal fluid samples retrieved via ST compared to dorsal and ventral cannula samples were also reported in Song et al. (2018). The relative abundance of both phyla contributed to 94% of total bacterial abundance in both ST and RC samples in that study. Interestingly, in the RC samples in the current study, the relative abundance of *Firmicutes* progressively increased whereas *Bacteroidetes* decreased from 0 to 12 h after feeding. Such patterns were not observed in ST samples. Further, changes in bacterial populations (classes 1 and 2; Supplementary Figures S1, S2) appear to follow changes in ruminal pH with time in the respective sampling method. Therefore, differences in pH and consequently bacterial populations between the two methods may be associated with the sampling location within the rumen.

Findings of the current study agree with those of Wirth et al. (2018) where the authors described the presence and composition of a core microbiota in the planktonic phase of cow’s rumen. Although a core microbiota existed in the rumen liquid phase, the relative abundance of the individual microbial populations varied and were influenced by sampling method, sampling time, and their interactions. Interestingly, the effect of sampling method was observed predominantly in *Bacteroidetes* members with a greater relative abundance for most of the bacterial genera in ST compared to RC ruminal fluid samples. Members of *Bacteroidetes* have been observed to be the first to respond or the most vulnerable to changes in ruminal environment induced by changes in feed composition and behavior (Pitta et al., 2018). Therefore, it is not surprising that these *Bacteroidetes* members were influenced both by an interaction between sampling time and sampling method and by sampling method itself. However, the most abundant populations of *Firmicutes*, such as unclassified *Lachnospiraceae*, unclassified *Ruminococcaceae*, and *Butyrivibrio*, showed a progressive increase in relative abundance with time in the RC samples.

Changes in metabolites produced by microbiota post-feeding has a greater in modulating microbial composition that overpowers variability between individual animals or effects of dietary composition (Shaani et al., 2018) indicating that fermentation activity also modulates microbial composition. Diurnal variations in bacterial populations were also observed by Lengowski et al. (2016), where they reported that feed intake and fermentation activity can lead to diurnal patterns in bacterial populations, mainly in the liquid fraction of rumen contents. These authors reported that feed, water, and salivary flow can affect the distribution of microbial populations such that bacteria, protozoa, and methanogens from the liquid phase can migrate to solid particles with an influx of feed and hence lead to changes in post-prandial copy numbers of individual microbial populations. While feed can influence the distribution of microbial populations in RC samples, saliva and water intake can be additional factors in the cranio-dorsal rumen leading to greater fluctuations in microbial diversity in postprandial ST samples with respect to ruminal pH, VFA, and bacterial populations. It may be inferred that the position of the ST in the rumen is critical and efforts to pass the probe beyond 200 cm depth into the ventral rumen provides a sample that is similar to RC samples.

There is an increasing interest in using ST to collect ruminal fluid for both fermentation and microbial analyses. In the present experiment, the length of time needed to perform ST sampling varied from 3 to 5 minutes, at each time point, and did not reduce daily DMI and milk yield of the animals enrolled in the experiment. This may indicate that, if done properly, ST sampling does not cause persistent stress in the animals. On the other hand, to make ST-derived samples similar to those of RC for pH, VFA concentrations, and bacterial populations, it is important to ensure that ST samples are collected from the ventral sac (Shen et al., 2012). While the liquid-only fraction is commonly used to make comparisons for fermentation parameters, both solid and liquid fractions should be considered for microbial analysis. Several papers that describe bacterial diversity dynamics have used primer pairs targeting the V1–V3 region of the 16S rRNA gene. Kittelmann et al. (2013) and Li et al. (2009) used the V1–V3 region of the 16S rRNA gene for bacterial diversity. Henderson et al. (2013) also used the V1-V3 region of the 16S rRNA gene but when 400 bp reads were considered, only the V1–V2 region was retained. For better recovery and consistency we used primers that targeted the V1–V2 region of the 16S rRNA gene. Because the same methodology was applied for all samples, we believe that differences observed between the two sampling methods were not attributable to differences in sample processing methods. In the present experiment, bacterial communities analyzed only in the liquid fraction were similar between RC and ST at pre-feed sampling but differed >2 h post-feeding. Nevertheless, a core microbiota was present across samples collected using both methods that was unique to each experimental cow. Studies should be done to better understand differences in bacterial communities between liquid and solid phases. In addition, exploring other non-invasive methods such as bolus or mouth swabs must be considered to better understand the distribution of microbial populations in the rumen of intensively managed dairy cows at different timepoints post-feeding.

## Conclusion

Results from the current study suggest that ruminal fluid samples collected through an oral ST (at 180 to 200 cm depth) are not representative of rumen pH, absolute values of VFA concentrations, or bacterial communities >2 h post-feeding when compared to samples of ruminal fluid collected through the rumen cannula. However, ST can be a feasible sampling technique if the purpose is to study molar proportions of VFA, protozoa counts, dH_2_ and ammonia concentration. In addition, as no differences were noted at the community level of ruminal bacteria, ruminal sampling using ST within the first 2 h after feeding may serve as a proxy for the liquid fraction of RC. In studies where large number of dairy cows must be screened for microbial analysis, ST may be the most suitable method for rumen sampling. Overall, our data indicate that there are substantial post-prandial differences in rumen fermentation variables and microbiota between samples collected using ST and RC techniques.

## Data Availability Statement

The bacterial raw sequences have been deposited in the NCBI Sequence Read Archive (SRA) database under BioProject accession number PRJNA630069.

## Ethics Statement

The animal study was reviewed and approved by The Pennsylvania State University Animal Care and Use Committee.

## Author Contributions

DP and AH hypothesis and experimental design. CA, SR, AM, MF, KN, JB, BV, MH, DP, and AH animal experiment and sampling. CA, SR, XC, JO, BV, and MH laboratory analysis. NI bioinformatic analysis. CA, MH, NI, DP, and AH manuscript preparation. All authors contributed to the article and approved the submitted version.

## Conflict of Interest

The authors declare that the research was conducted in the absence of any commercial or financial relationships that could be construed as a potential conflict of interest.

## Abbreviations

ASV: amplicon sequence variants
PCoA: principal co-ordinate analysis
PERMANOVA: permutational multivariate ANOVA
VFA: volatile fatty acid.
