# Supplementary figures and images for: Comparison of Two Sampling Techniques for Evaluating Ruminal Fermentation and Microbiota in the Planktonic Phase of Rumen Digesta in Dairy Cows
Source: Front Microbiol. 2020 Dec 23;11:618032. doi: 10.3389/fmicb.2020.618032 (PMC7785721; doi:10.3389/fmicb.2020.618032)

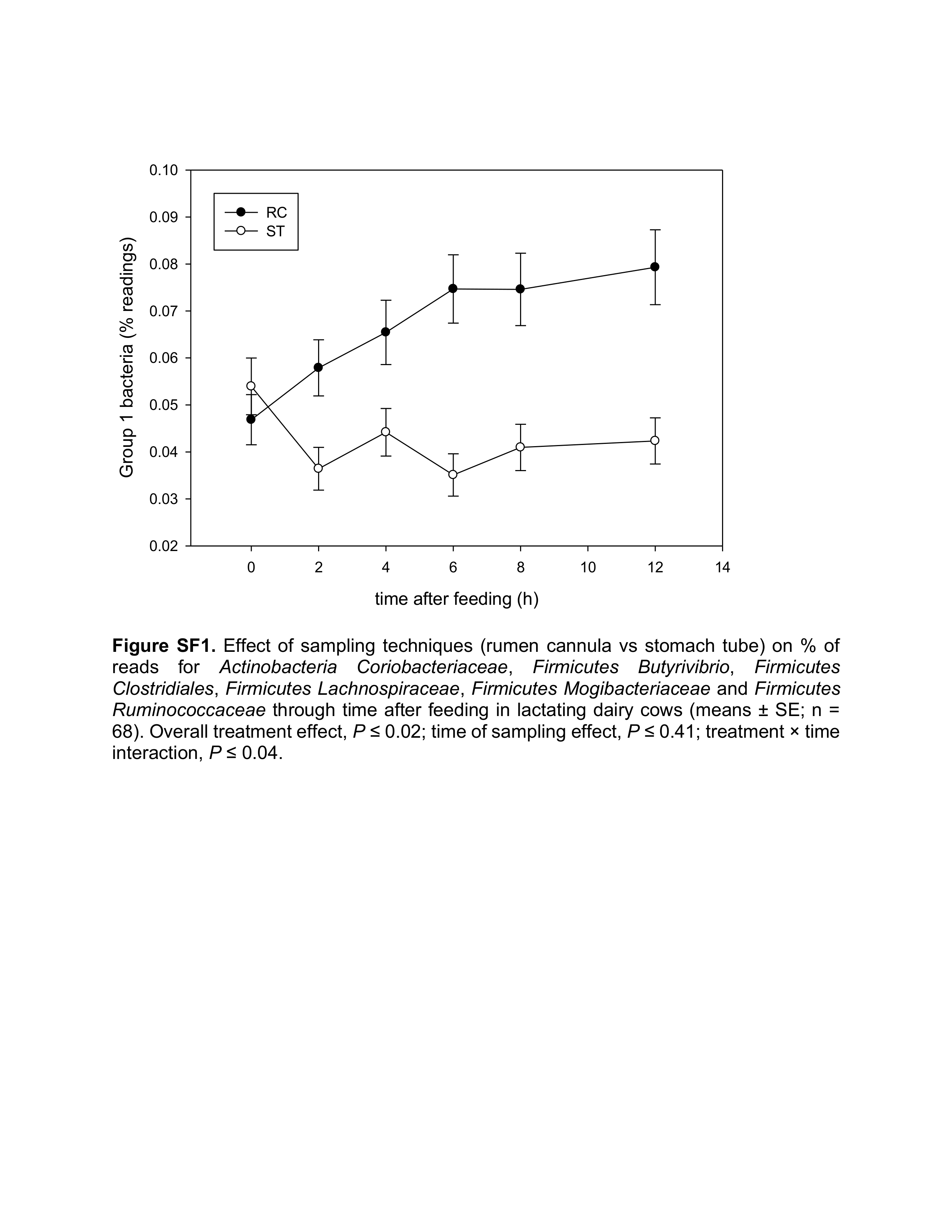

Supplement: Supplementary file 1 [file Image_1.tif]

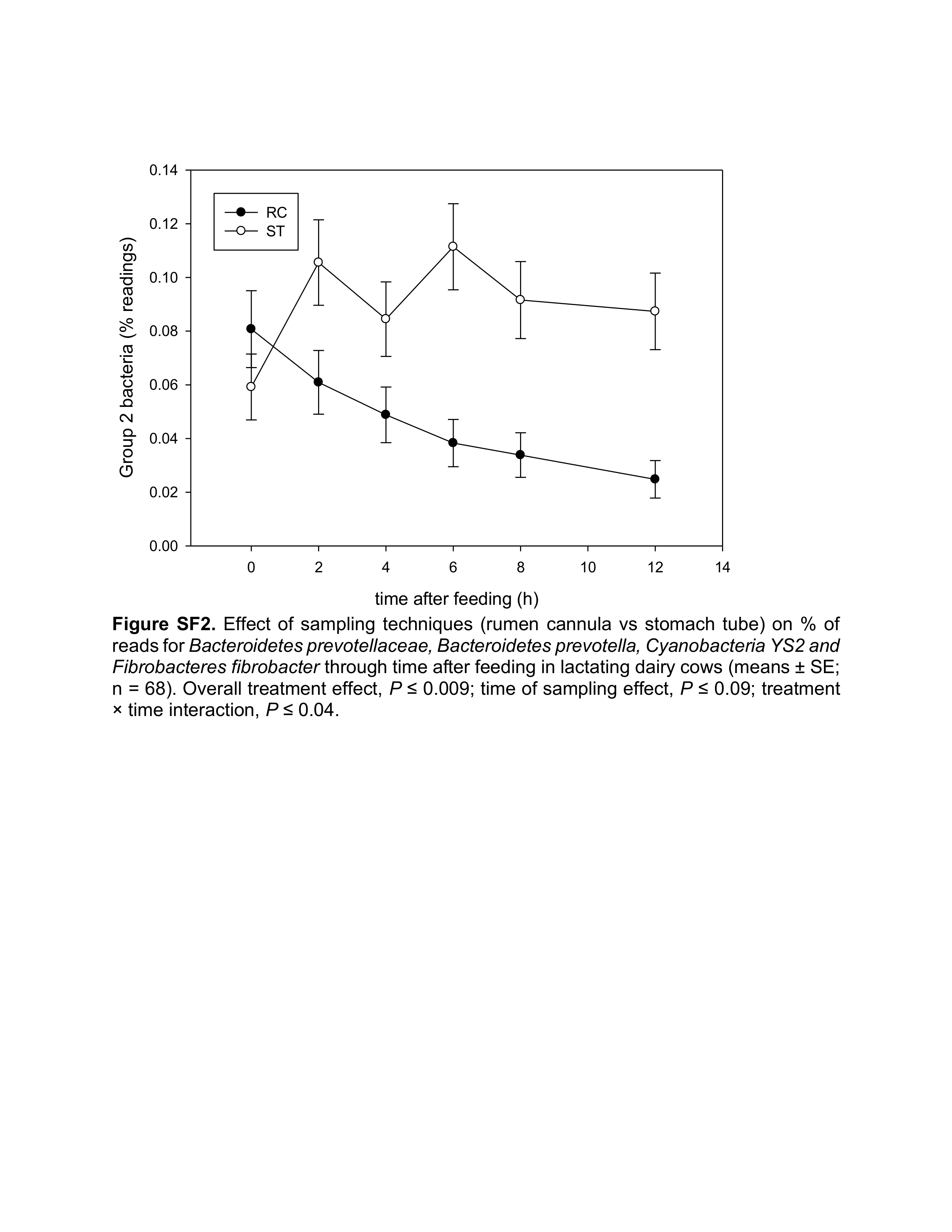

Supplement: Supplementary file 2 [file Image_2.tif]
